# Sports and Kinetic Visual Acuity

**DOI:** 10.14789/jmj.JMJ22-0019-R

**Published:** 2022-08-02

**Authors:** KEISUKE SAWAKI, YOSHIMITSU KOHMURA, KAZUHIRO AOKI, MITSURU NAKAMURA, SHIGEKI MURAKAMI, YOSHIO SUZUKI

**Affiliations:** 1 Faculty of Health and Sports Science, Juntendo University, Chiba, Japan; 1 Faculty of Health and Sports Science, Juntendo University, Chiba, Japan; 2 Murakami Eye Clinic, Kumamoto, Japan; 2 Murakami Eye Clinic, Kumamoto, Japan

**Keywords:** kinetic vision acuity, dynamic visual acuity, athletics

## Abstract

Kinetic vision acuity (KVA) is an index developed in Japan that refers to the capacity to recognize a moving object that moves back and forth against the observer. This review outlines the history of KVA and studies on KVA conducted at the Faculty of Health and Sports Science of Juntendo University, i.e. characteristics of KVA in athletes, factors associated with KVA, sports and age-dependent decline of KVA, and effects of docosahexaenoic acid (DHA) and astaxanthin on KVA. KVA was defined in the early 1960s, and the measurement device was invented in 1968. Studies at the Faculty of Health and Sports Science began in the 1990s. In track-and-field athletics and skeleton, a winter downhill event, higher-ranked athletes had higher KVA than lower-ranked athletes. Although KVA cannot be predicted from static visual acuity or reaction time, a significant correlation was found between KVA and the peak latency of visual-evoked potentials. KVA could not be improved by training and did not change between age of 8 and 17 years. In contrast, habitual practice in kendo may inhibit the age-dependent decline in KVA. DHA may also improve KVA in subjects with low KVA; however, astaxanthin did not improve KVA.

## Introduction

The ability to recognize a moving object differs from that to recognize a stationary object. Kinetic vision acuity (KVA) is an index developed in Japan to measure the ability to recognize moving objects that move back and forth against the observer. Since the 1990s, studies on the relationship between KVA and sports have been conducted at Juntendo University Faculty of Health and Sports Science. However, the overall results remain obscure because they were published in various journals mostly in Japanese. Therefore, this review outlines the history of KVA developed in Japan and studies conducted at the Faculty of Health and Sports Science of Juntendo University, i.e. characteristics of KVA in athletes, factors associated with KVA, sports and age-dependent decline of KVA, and effects of docosahexaenoic acid (DHA) and astaxanthin on KVA.

## Brief history of KVA

The representative capacity to recognize moving objects includes dynamic visual acuity (DVA) and KVA. DVA is the ability to recognize objects moving in all directions, including left, right, up, and down when the object, the observer, or both, are moving, at an equal distance from the observer. In 1949, Ludvigh showed that stationary objects are more clearly visible than moving ones^1)^. Moreover, in 1953, he reported that the DVA decreased as the angular velocity of the test object moving in the horizontal plane increased from 10 to 170 deg/sec. There was also a significant difference in DVA in subjects with almost the same static visual acuity (SVA)^2)^. A recent review summarizes the characteristics of DVA in comparison with SVA^3)^. In Japan, Kowa (Nagoya, Japan) launched the first DVA-measuring device HI-10 in 1998. The device had been mainly used under the guidance of the Japan Sports Vision Association although it is no longer manufactured and sold.

**Figure 1:**
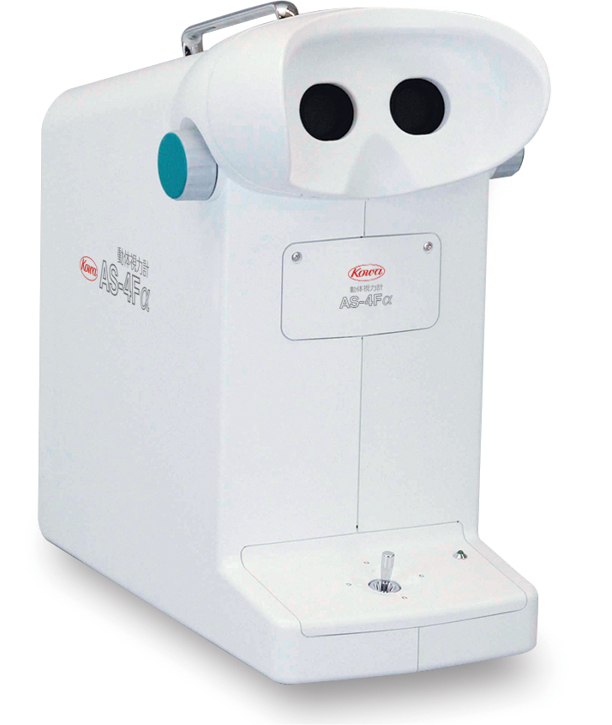
Kinetic visual acuity tester AS-4Fα (Kowa, Nagoya, Japan)

**Figure 2:**
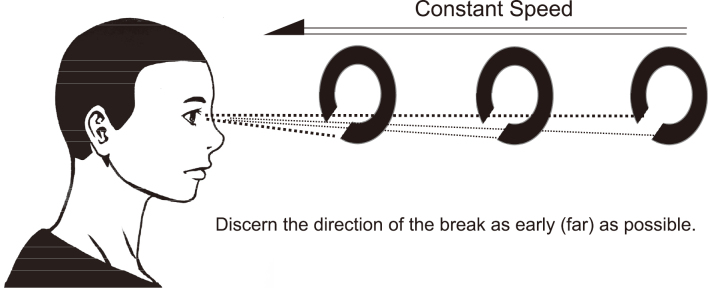
Schema to measure kinetic visual acuity. A Landolt ring is approaching from front at a constant speed. The observer discerns the direction of the break as early (far) as possible. This ability is measured as kinetic visual acuity.

Hagino et al. of Nagoya University began vigorous research on the ability to recognize moving objects in the 1950s^4)^. Suzumura defined KVA as the ability to recognize an object moving back and forth in the distance against the observer^4, 5)^. Suzumura invented a device to measure KVA in 1968^6)^. The device, AS-4A, was launched by Kowa (Nagoya, Japan) in 1966 and has been mainly used in Japan as a tool for driving education for automobile drivers. Currently, the AS-4Fα (Figure 1), the successor to the AS-4A (Kowa, Nagoya, Japan), is available, which identifies KVA as the ability to recognize the Landolt ring approaching the observer from 50 m from the front at 30 km/h (Figure 2).

To recognize a moving object accurately, it is necessary to quickly catch the object near the central fossa, adjust it, and bring it into focus. Moving objects are usually more difficult to see clearly than stationary ones; therefore, KVA does not exceed SVA. Suzumura has clarified that (1) KVA decreases as the speed of the target increases, (2) individual variability in KVA is large, (3) decline in KVA cannot be perceived, and (4) decline in KVA is unrelated to SVA^7)^.

DVA is widely used in Europe and the United States, whereas KVA is mainly used in Japan. In PubMed, search results for “dynamic visual acuity” included 349 papers, 266 of which were published in 2001 or later. Meanwhile, search results for “kinetic visual acuity” included 31 papers, only 5 of which were published in 2001 or later (searched on February 2, 2022). The large difference in the number of papers can be attributed to KVA being developed in Japan and many previously published studies being in Japanese.

## KVA of athletes

In 1974, Sanderson et al. reported a significant correlation between DVA and ball-catching performance and an absence of significant correlation between DVA and SVA^8)^. In healthy young adults, those who engage in sports activities were found to have better DVA than those who do not^9)^.

In Japan, the relationship between KVA and the batting ability of baseball players was reported at the annual meeting of the Japanese Society of Physical Education in 1971^10)^. However, there were no reports on the relationship of KVA with sports for the next 20 years. In 1992, Ishigaki et al. reported that in top-level Japanese basketball, volleyball, soccer, and baseball players, athletes with a higher competitive ability also had higher KVA^11)^. In 1995, people who played tennis continuously for more than 7 years were reported to have better KVA than those with no sports history^12)^. In addition, at the 1996 annual meeting Japan Society of Physical Education, Health and Sport Sciences, a professional baseball team case study reported that KVA was higher in regular players than in second-unit players when they joined the team^13)^.

Studies on KVA at Juntendo University’s School of Sports and Health Science began in 1996 with the DHA study by Sawaki & Yoshigi et al. described in “Effects of DHA and astaxanthin on KVA” below. Sakuma et al. investigated the SVA and KVA of male college track-and-field athletes and found that both SVA and KVA were higher in athletes engaged in steeplechase, jump, and mixed events than in short-, middle-, and long-distance runners and throwers and that SVA and KVA are higher in athletes with higher competitive ability^14)^. Aoki et al. investigated elite male pole vaulters and long jumpers and reported that there was no difference in KVA between pole vaulters and long jumpers and that the top competitive pole vaulters had higher SVA and KVA than the bottom pole vaulters^15)^. In addition, Inoue et al. compared the skeleton athletes competing in international sledding competitions and lower group competing in the Japanese National Championships to find that KVA was significantly higher in the upper group than in the lower group, whereas no difference was seen in SVA^16)^.

## Factors associated with KVA

Sado et al. examined the SVA and KVA of freshmen of the Faculty of Health and Sports Health Science whose SVA was 1.0 or higher^17)^. The number of subjects was 92 (78 men and 14 women aged between 18 and 20 years). SVA was greater than 1.0, whereas KVA ranged from 0.12 to 1.30. KVA could not be estimated from SVA although there was a weak but significant positive correlation between SVA and KVA (r = 0.486 in the right eye, r = 0.490 in the left eye, and r = 0.384 in both eyes)^17)^.

Kohmura et al. determined the association between KVA and reaction time. The reaction time, often used in sports science, reflects the ability to identify and respond quickly to visual cues. However, the correlation between KVA and reaction time was not apparent^18, 19)^. Yoshigi et al. also analyzed the relationship between KVA and each component of visual-evoked potential and found a significant correlation between KVA and the peak latency of visual-evoked potentials; the better the KVA, the shorter the peak latency^20)^.

Studies have been reported on the effects of training on KVA in baseball players. Kohmura et al. conducted an 8-week training experiment wherein college baseball players were trained to look at the ball at the batter’s box or to use commercially available software for visual function training. KVA was not improved by either training although some effects on visual function were observed^21)^. A subsequent study with junior high school students also revealed that training did not improve KVA^22)^.

Kohmura et al. studied 867 men and women aged 8-17 years and reported that DVA developed gradually with age, but KVA did not change from age 8 to 17 years^23)^. Therefore, KVA is expected to reach almost the same level as adults by the age of 8 years^23)^.

As described above, KVA has a large individual variation, may not be improved by training, and matures up to 8 years old, KVA could be available for finding talent in the junior period in sports.

## Sports and age-related decline of KVA

Nakamura et al. compared young and elderly kendo players (n = 30) with age-matched nonathletes (n = 30) and found that KVA significantly decreased with age, whereas contrast sensitivity, eye movement, and instantaneous visual acuity were not affected by age or exercise habits^24)^. At the same time, kendo players had a higher KVA than nonathletes (p < 0.01) when comparing ten young subjects each^24)^. Age-related decline in KVA has also been observed in young and middle-aged soccer players (n = 44) and in nonathletes of the same age group (n = 45)^25)^. Recently, Kudo et al. reported kendo players (n = 41; 35.4 ± 15.7 years, range 19-65 years) had significantly higher KVA (p < 0.01) than nonexercisers (n = 65; 38.1 ± 17.1 years, range 19-71 years)^26)^.

Therefore, KVA declines with age, and training habits to gaze at moving objects, such as kendo, may suppress the age-related decline. However, the effect of sports remains unclear.

## Effects of DHA and astaxanthin on KVA

DHA is an n-3 fatty acid found in the retina and the brain’s gray matter^27)^. In 1993, artificial milk containing fish oil was reported to improve visual acuity in preterm infants up to the fourth month of life^28)^. This research led to the study of the effect of DHA on KVA.

In 1997, Sawaki et al. reported a randomized, double-blind study to examine the effects of DHA (1,500 mg/d) for 35 consecutive days in 44 male collegiate athletes and baseball players with an SVA of 1.0 or higher^29)^. DHA intake significantly increased KVA from 0.87 ± 0.24 to 0.97 ± 0.21 (p < 0.01) but did not change SVA. The improvement was greater in participants with low KVA before intervention. No change was observed in the control group (equal amounts of soybean oil)^29)^.

To confirm the results of Sawaki et al.^29)^, Ishigaki et al. conducted a double-blind study of DHA (1,500 mg/d) for 30 consecutive days in 20 university long-distance runners and 12 fencing players^30)^. The mean SVA significantly increased from 1.21 to 1.32 in the DHA group (p < 0.05), whereas there was no significant change in the placebo (safflower oil) group. The mean KVA improved from 0.65 to 0.69 in the DHA group, but not significant^30)^.

Subsequently, another study was conducted with 55 college athletes as participants: 8 tennis players, 18 volleyball players, 15 track-and-field athletes, and 14 baseball players (47 men and 8 women)^31)^. A total of 28 participants did not take orthoptics, whereas 25 and 2 of them used contact lenses and glasses, respectively. DHA (1,500 mg/d) was given for 35 consecutive days, and their visual acuity was measured before and after the intervention. There were no significant differences in SVA and KVA between the groups (25 and 26 in the DHA and control groups, respectively). Low-contrast visual acuity improved in the DHA group but not in the control group (p < 0.05). When preintervention KVA was stratified by LogMAR = 0.3 (decimal acuity 0.5), subjects with lower preintervention KVA improved in the DHA group (n = 10) but not in the control group (n = 13; between-group comparison, p < 0.05). Therefore, DHA may improve KVA in subjects with low KVA^31)^.

Sawaki et al. also determined the effect of astaxanthin. A total of 18 male collegiate handball players ingested astaxanthin (6 mg/d, n = 9) or placebo (n = 9) for 4 weeks. No significant improvement was observed on SVA, KVA, or KVA/SVA^32)^.

## Summary

This review aimed to outline the history of KVA followed by the studies on KVA conducted at Juntendo University Faculty of Health and Sports Science. Suzumura defined KVA as the ability to recognize an object moving back and forth in the distance against the observer, and invented a device to measure KVA. Studies on KVA has been conducted at Juntendo University Faculty of Health and Sports Science since 1990s. Athletes who require great attention to moving objects have a higher KVA. Further, athletes at higher levels of competition have higher KVA. The relationship of KVA to simple reaction time is unclear. In contrast, some studies suggest a correlation between KVA and peak latency of visual-evoked potentials. However, the mechanism to determine KVA requires more investigations. Alternatively, KVA represents a potential tool for discovering junior sports talent as it cannot be improved by training after 8 years old. Moreover, KVA declines with age although habitual exercise that requires a clear, quick vision of a moving object may suppress the age-related decline of KVA. In addition, DHA may improve KVA in subjects with low KVA. As described above, KVA is a unique visual acuity distinct from SVA. However, further studies are necessary to determine the practical application of KVA.

## Funding

The authors received no financial support for the research.

## Author contributions

K.S. conceptualized this review. Y.K., K.A., and Y.S. searched and screened literatures to review.

Y.K., K.A., M.N., S.M., and Y.S. summarized the literatures. Y.K. and Y.S. wrote the draft manuscript. K.S., Y.K., and Y.S. reviewed and edit the manuscript. All authors have read and agreed to the published version of the manuscript.

## Conflicts of interest statement

The authors declare that there are no conflicts of interest.
